# RACE-trial: neoadjuvant radiochemotherapy versus chemotherapy for patients with locally advanced, potentially resectable adenocarcinoma of the gastroesophageal junction - a randomized phase III joint study of the AIO, ARO and DGAV

**DOI:** 10.1186/s12885-020-07388-x

**Published:** 2020-09-15

**Authors:** Sylvie Lorenzen, Alexander Biederstädt, Ulrich Ronellenfitsch, Christoph Reißfelder, Stefan Mönig, Frederik Wenz, Claudia Pauligk, Martin Walker, Salah-Eddin Al-Batran, Bernhard Haller, Ralf-Dieter Hofheinz

**Affiliations:** 1 Department of Internal Medicine III (Haematology/Medical Oncology), Technical University Munich, Ismaningerstr. 22, 81675 Munich, Germany; 2 Department of Visceral, Vascular and Endocrine Surgery, University Hospital Halle (Saale), Halle (Saale), Germany; 3 Department of Surgery, Medical Faculty Mannheim, Heidelberg University, Mannheim, Germany; 4 Visceral Surgery Department, Geneva University Hospital, Geneva, Switzerland; 5 University of Freiburg, Freiburg, Germany; 6 Institute of Clinical Cancer Research IKF at Northwest hospital, UCT University Cancer Center, Frankfurt am Main, Germany; 7 Institute for Medical Statistics and Epidemiology, Technical University Munich, Munich, Germany; 8 Interdisciplinary Tumour Centre Mannheim, University Medicine of Mannheim, Mannheim, Germany

**Keywords:** Locally advanced adenocarcinoma of the gastroesophageal junction, Neoadjuvant radiochemotherapy, Perioperative chemotherapy, FLOT

## Abstract

**Background:**

Despite obvious advances over the last decades, locally advanced adenocarcinomas of the gastroesophageal junction (GEJ) still carry a dismal prognosis with overall 5-year survival rates of less than 50% even when using modern optimized treatment protocols such as perioperative chemotherapy based on the FLOT regimen or radiochemotherapy. Therefore the question remains whether neoadjuvant chemotherapy or neoadjuvant radiochemotherapy is eliciting the best results in patients with GEJ cancer. Hence, an adequately powered multicentre trial comparing both therapeutic strategies is clearly warranted.

**Methods:**

The RACE trial is a an investigator initiated multicenter, prospective, randomized, stratified phase III clinical trial and seeks to investigate the role of preoperative induction chemotherapy (2 cycles of FLOT: 5-FU, leucovorin, oxaliplatin, docetaxel) with subsequent preoperative radiochemotherapy (oxaliplatin weekly, 5-FU plus concurrent fractioned radiotherapy to a dose of 45 Gy) compared to preoperative chemotherapy alone (4 cycles of FLOT), both followed by resection and postoperative completion of chemotherapy (4 cycles of FLOT), in the treatment of locally advanced, potentially resectable adenocarcinoma of the gastroesophageal junction. Patients with cT3–4, any N, M0 or cT2 N+, M0 adenocarcinoma of the GEJ are eligible for inclusion. The RACE trial aims to enrol 340 patients to be allocated to both treatment arms in a 1:1 ratio stratified by tumour site. The primary endpoint of the trial is progression-free survival assessed with follow-up of maximum 60 months. Secondary endpoints include overall survival, R0 resection rate, number of harvested lymph nodes, site of tumour relapse, perioperative morbidity and mortality, safety and toxicity and quality of life.

**Discussion:**

The RACE trial compares induction chemotherapy with FLOT followed by preoperative oxaliplatin and 5-Fluorouracil-based chemoradiation versus preoperative chemotherapy with FLOT alone, both followed by surgery and postoperative completion of FLOT chemotherapy in the treatment of locally advanced, non-metastatic adenocarcinoma of the GEJ. The trial aims to show superiority of the combined chemotherapy/radiochemotherapy treatment, assessed by progression-free survival, over perioperative chemotherapy alone.

**Trial registration:**

ClinicalTrials.gov; NCT04375605; Registered 4th May 2020;

## Background

While overall gastric cancer incidence is decreasing, the incidence of adenocarcinoma of the GEJ has increased in North America and Western European countries [1, 2]. This shift of tumour location towards the GEJ, as well as the histological trend from squamous cell carcinoma (SCC) to adenocarcinoma demands adaptations for therapeutic recommendations in Western populations.

With disease symptoms appearing late in the course of the disease, most of the patients present with locally advanced or metastatic disease at first diagnosis resulting in dismal 5-year survival rates of 20–30% [3]. Surgery remains the only form of curative treatment for non-metastatic gastroesophageal cancer but is associated with high rates of local or distant recurrence.

Intensive work has been done to increase cure rates in patients with locally advanced GEJ cancers utilising chemotherapy and radiotherapy in the neoadjuvant and adjuvant treatment setting. In patients with resectable tumours, there is evidence supporting the use of both, perioperative chemotherapy and preoperative radiochemotherapy; however, there is an ongoing debate what is the best treatment option and trials thus far have proven inconclusive to favour one approach over the other.

According to the most recent German S3-guideline for the treatment of gastroesophageal cancer, both, perioperative chemotherapy and preoperative radiochemotherapy are regarded as reasonable treatment options [4]. In recent years, evidence has emerged suggesting a beneficial role of neoadjuvant chemoradiation compared to preoperative chemotherapy alone.

Perioperative chemotherapy with epirubicin, cisplatin and infusional 5-fluorouracil (ECF) [5] and cisplatin and 5-fluorouracil (CF) [6] has been demonstrated in two randomized trials to improve 5-year survival rates by 13 and 14%, respectively over surgery alone in gastric and GEJ cancers of stage ≥II. These results support the use of perioperative chemotherapy as a standard of care for resectable esophagogastric adenocarcinoma. However, both trials included gastric tumours, which makes interpretation of the results challenging. Moreover, the 5-year survival rate of clearly less than 50% in both trials remains unsatisfactory and warrants the search for further potent drugs with cytotoxic or molecularly targeted mechanism of action, such as taxanes, platin alternatives or monoclonal antibodies.

In search of an optimized preoperative chemotherapy regimen, the Arbeitsgemeinschaft Internistische Onkologie (AIO) has conducted a phase III trial comparing FLOT with ECF in the neoadjuvant treatment of patients with resectable esophagogastric adenocarcinoma (FLOT4 trial) [7]. FLOT improved OS (median OS, 35 months with ECX/ECF vs. 50 months with FLOT; HR 0.77 [0.63–0.94]; *p* = 0.012). 3y OS rate was 48% with ECF/ECX and 57% with FLOT. FLOT also improved PFS (mPFS, 18 months with ECX/ECF vs. 30 months with FLOT; HR 0.75 [0.62–0.91]; *p* = 0.004). Perioperative complications were 50% with ECF/ECX and 51% with FLOT. 30- and 90-day mortality was 3 and 8% with ECF/ECX and 2 and 5% with FLOT. Regarding toxicities, in the FLOT group, all patients had some type of toxicity (57.1% reporting ≥3 symptoms), however, side effects were generally manageable. The relative effect from FLOT was observed in all subgroups, including elderly patients and signet cell tumours, and was particularly pronounced in AEG type 1 tumours (HR 0.60), Barrett tumours (HR 0.62), T1/2 tumours (HR 0.66) and nodal negative tumours (HR 0.64). FLOT was found to be superior to ECF/ECX in all relevant subgroups and is nowadays widely regarded as the perioperative chemotherapy regimen of choice in esophagogastric adenocarcinoma.

Alternatively, the strategy of neoadjuvant radiochemotherapy has been implemented due to the results of the CROSS trial, in which van Hagen and colleagues randomly assigned 368 patients with esophageal cancer (23% SCC and 75% adenocarcinoma) into either a neoadjuvant radiochemotherapy regimen, based on weekly carboplatin and paclitaxel followed by surgery, or surgery alone [8]. Neoadjuvant radiochemotherapy improved overall survival (median 49 vs. 24 months; *p* = 0.003; HR 0.657) with comparable postoperative morbidity and mortality rates of 46 and 4%, respectively. The benefit of neoadjuvant radiochemotherapy on survival was especially seen for patients with SCC and to a lesser extent for patients with adenocarcinoma. Toxicity of radiochemotherapy was low in terms of hematologic and non-hematologic side effects.

With regard to the radiochemotherapy protocol, concurrent radiochemotherapy regimens generally use a combination of cisplatin and 5-fluorouracil or carboplatin and paclitaxel. However, it could be shown that a combination of oxaliplatin and continuous infusion of 5-fluorouracil together with radiotherapy is also well tolerated and efficacious for localized esophageal cancer [9, 10].

With promising results from both, the CROSS and the FLOT4 trial, the question remains whether neoadjuvant chemotherapy or neoadjuvant radiochemotherapy is eliciting the best results in patients with GEJ cancer.

A benefit for neoadjuvant radiochemotherapy vs. chemotherapy alone has been suggested from indirect comparisons in meta-analyses but could not be clearly demonstrated in direct comparisons [11, 12]. Two studies directly compared the outcome of patients receiving either perioperative chemotherapy or neoadjuvant radiochemotherapy [13, 14]. There was no significant difference in survival between the two arms in either of the clinical studies, even though a trend for improved survival was noted for radiochemotherapy. Of note, the clinical implications of all of these studies are limited due to small sample size [12, 13, 15].

In addition, all of the trials have been performed in studies with mixed patient cohorts and before the FLOT regimen and the CROSS protocol as most effective neoadjuvant treatment approaches for GEJ cancers became standard of care.

Since both strategies, radiochemotherapy and perioperative chemotherapy provide significant gains in survival, we hypothesize that adding radiochemotherapy to the FLOT regimen will achieve even greater survival gains in the similar patient population.

The RACE trial addresses the question if preoperative FLOT induction chemotherapy followed by preoperative radiochemotherapy and postoperative completion FLOT chemotherapy is superior to perioperative FLOT chemotherapy alone in patients with adenocarcinoma of the gastroesophageal junction undergoing adequate oncological surgery.

## Methods/design

**Fig. 1:**
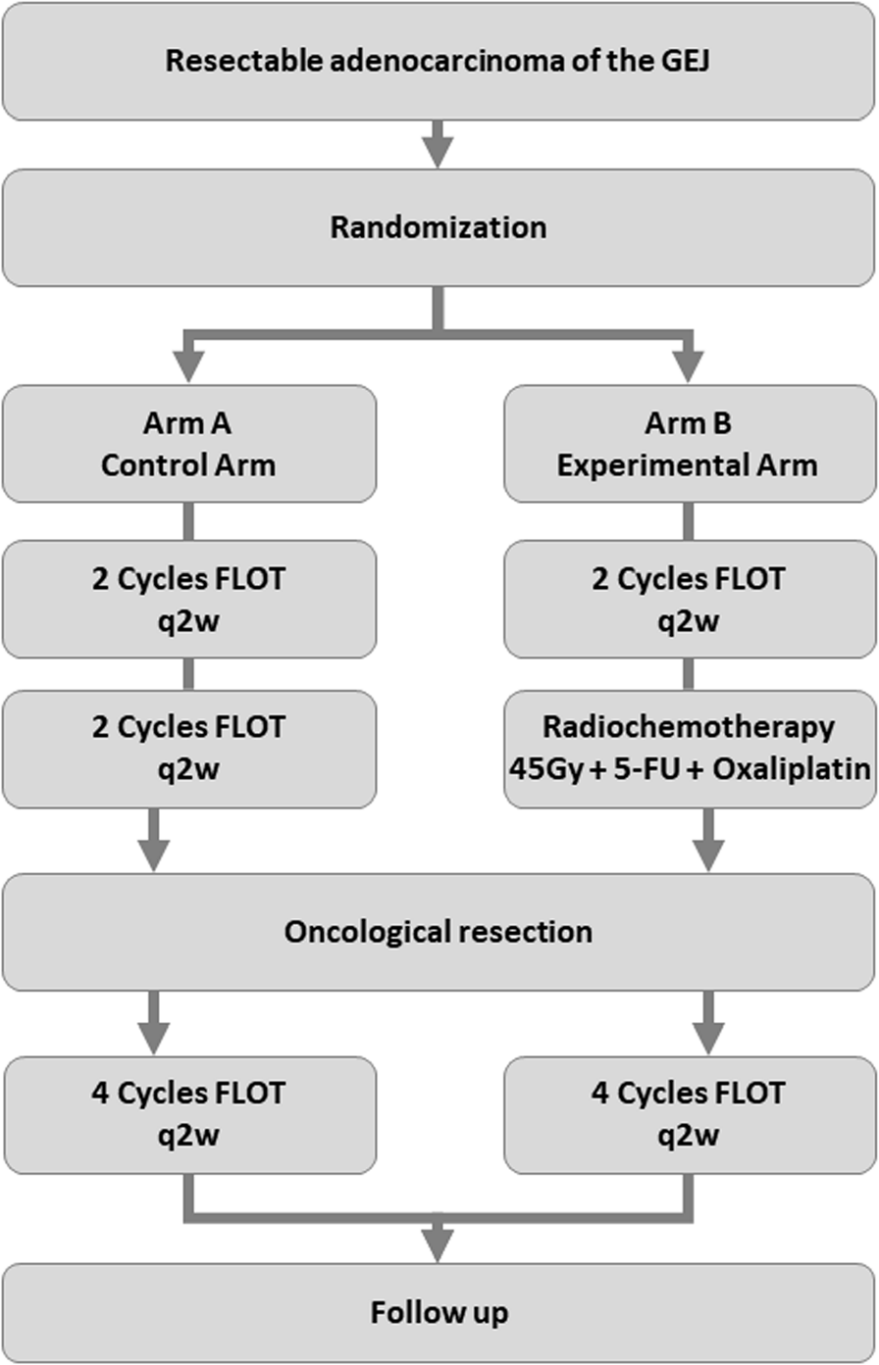
Trial schema. Patients with resectable adenocarcinoma of the gastroesophageal junction are randomized to either receive 4 cycle of neoadjuvant FLOT chemotherapy (control arm) or 2 cycles of neoadjuvant FLOT induction chemotherapy followed by neoadjuvant chemoradiation (experimental arm) to 45 Gy with concurrent 5-FU/oxaliplatin-based chemotherapy, both followed by oncological resection and completion of chemotherapy with four further cycles of FLOT

The RACE trial is an investigator initiated multicentre, prospective, randomized, stratified phase III clinical trial and is financially supported by Deutsche Krebshilfe e.V. (German Cancer Aid). Eligible patients will be randomly allocated to one of two treatment groups, i.e. preoperative chemotherapy or preoperative chemotherapy with subsequent preoperative radiochemotherapy, both followed by resection and postoperative completion of chemotherapy (Fig. 1.). The protocol was submitted to and has been approved by the Ethics Committee II of the University of Heidelberg on September 24th 2019, as well as individual institutional ethics committees. The primary objective of the trial is to demonstrate whether the addition of preoperative chemoradiation to perioperative chemotherapy prolongs progression-free survival compared to perioperative chemotherapy alone. The RACE trial aims to enrol 340 patients across 40 trial sites randomized in a 1:1 ratio and stratified by primary tumour site (AEG type I vs AEG type II/III). All trial sites are highly experienced in the treatment of patients with gastrointestinal malignancies including esophageal surgery. Written informed consent will be obtained from all participating trial subjects.

### Target population

Patients are deemed eligible for trial participating with histologically confirmed diagnosis of locally advanced, potentially resectable non-metastatic adenocarcinoma of the gastroesophageal junction (AEG type I-III). Patients need to provide written informed consent before trial enrolment and must meet all of the following inclusion and exclusion criteria.

### Inclusion criteria

1. Histologically proven, locally advanced and potentially resectable adenocarcinoma of the gastroesophageal junction (GEJ) (AEG I- III): cT3–4, any N, M0 or cT2 N+, M0 (AJCC 8th edition)
2. Patients must be candidates for potential curative resection
3. ECOG performance status 0–1
4. Age ≥ 18 years
5. Adequate hematologic function with absolute neutrophil count (ANC) ≥ 1.5 × 10^9^/l, platelets ≥100 × 10^9^/l and haemoglobin ≥9.0 mg/dl
6. INR < 1.5 and aPTT< 1.5 x upper limit of normal (ULN)
7. Adequate liver function (serum transaminases ≤2.5 x ULN and total bilirubin ≤1.5 x ULN)
8. Adequate renal function (serum creatinine ≤1.5 x ULN)
9. QTc interval (Bazett) ≤ 440 ms
10. Written informed consent obtained before randomization
11. Negative pregnancy test for women of childbearing potential. Males and females of reproductive potential must agree to practice highly effective contraceptive measures.

### Exclusion criteria

Any of the following renders patients ineligible to participate in this trial:

1. Evidence of metastatic disease
2. Past or current history (within the last 5 years) of other malignancies.
3. Evidence of peripheral sensory neuropathy > grade 1 according to CTCAE version 4.03
4. Significant underlying medical conditions that may be aggravated by the study treatment or are not controlled
5. Pregnant or lactating females
6. Patients medically unfit for chemotherapy and radiotherapy
7. Patients receiving any immunotherapy, cytotoxic chemotherapy or radiotherapy other than defined by the protocol
8. Known hypersensitivity against 5-FU, leucovorin, oxaliplatin or docetaxel
9. Other known contraindications against 5-FU, leucovorin, oxaliplatin, or docetaxel
10. Clinically significant coronary heart disease, cardiomyopathy or congestive heart failure, NYHA III-IV
11. Clinically significant valvular defect
12. Other severe internal disease or acute infection
13. Peripheral polyneuropathy > NCI Grade II according to CTCAE version 4.03
14. Chronic inflammatory bowel disease

### Study treatment

#### Perioperative chemotherapy (arm A)

Patients randomized into the perioperative chemotherapy arm receive 4 cycles of neoadjuvant chemotherapy with FLOT every 2 weeks (5-FU 2600 mg/m^2^ d1, leucovorin 200 mg/m^2^ d1, oxaliplatin 85 mg/m^2^ d1, docetaxel 50 mg/m^2^ d1) followed by surgical resection 4–6 weeks after day 1 of the last cycle of neoadjuvant therapy. 6–12 weeks after surgery adjuvant chemotherapy starts with 4 cycles of FLOT (total treatment period 25–32 weeks).

#### Neoadjuvant induction chemotherapy followed by chemoradiation (arm B)

Trial subjects in the investigational arm receive 2 cycles of neoadjuvant induction chemotherapy with FLOT (doses as above) every 2 weeks (4 weeks of therapy) followed by radiochemotherapy beginning at day 21 after day one of the last cycle of chemotherapy. Radiochemotherapy consists of oxaliplatin 45 mg/m^2^ weekly (d1, 8, 15, 22, 29) and continuous infusional 5-FU 225 mg/m^2^ plus concurrent radiotherapy given in 5/week fractions with 1.8 Gy to a dose of 45 Gy over 5 weeks. Resection is performed 4–6 weeks after last treatment with chemotherapy / radiation. Adjuvant treatment starts 6–12 weeks after surgery and consists of 4 cycles of FLOT (total treatment period of 26–33 weeks).

#### Surgery (both arms)

Four–six weeks after completion of neoadjuvant treatment, patients in both arms will be scheduled for operation.

#### Esophageal resection and extent of lymphadenectomy

AEG type I tumours are treated by resection of the proximal stomach and transthoracic esophagectomy with 2-field lymphadenectomy.

AEG type II and III tumours are treated by gastrectomy with transhiatal resection of the distal oesophagus and transhiatal lymphadenectomy of the lower mediastinum.

For all tumour types, abdominal lymphadenectomy is performed as D2 dissection. The minimum number of lymph nodes to be harvested and pathologically analysed is 16, with 25 lymph nodes recommended.

#### Surgical reconstruction

After transthoracic esophagectomy, reconstruction is preferably performed by an intrathoracic anastomosis with a gastric conduit, alternatively by cervical anastomosis or colon interposition. After gastrectomy and transhiatal resection of the distal oesophagus, reconstruction is carried out by esophagojejunostomy.

### Study objectives and endpoints

#### Primary endpoint

The primary objective is to investigate whether neoadjuvant induction chemotherapy with FLOT followed by radiochemotherapy is superior to perioperative chemotherapy alone in the treatment of resectable GEJ adenocarcinoma in patients undergoing oncologically adequate surgery (D2 resection and appropriate mediastinal lymphadenectomy). The primary endpoint is progression-free survival, recorded as time from randomization until disease-progression, disease recurrence after surgery or death of any cause. Moreover, incomplete (R1 or R2) resection as well as irresectability will be recorded as PFS events. Patients lost to follow up will be censored to the date of last assessment without any such event. Patients will be followed up for a maximum of 60 months after randomization.

#### Secondary endpoints

- Overall survival, including survival rates after 1, 3 and 5 years: Overall survival will be measured as time from randomization to death of any cause or last observation.
- R0 resection rate
- Number of harvested lymph nodes
- Site of tumour relapse (locoregional, peritoneal, distant, or a combination of multiple sites)
- Perioperative morbidity and mortality rate
- Safety and toxicity by NCI CTC criteria
- Quality of life (QoL) by using the EORTC QLQ-C30 and the esophagogastric module OG25

### Data collection and follow-up

#### Assessments at screening

Screening assessments need to be completed within 4 weeks prior to randomization. Once diagnosis of gastroesophageal adenocarcinoma is confirmed, pre-therapeutic work up includes an electrocardiogram as well as assessment of tumour localisation and size by esophagogastroduodenoscopy and assessment of tumour infiltration and locoregional lymph node involvement based on endoscopic sonography. Computed tomography of the chest, abdomen and pelvis is performed to exclude metastatic disease. For patients with suspected peritoneal tumour seeding, a diagnostic laparoscopy is performed. Within 2 weeks prior to the start of treatment, patients are further evaluated based on their medical history, a physical examination including body weight, height, vital signs and ECOG performance status. Laboratory testing includes a differential blood count and standard clinical chemistry. Patients meeting all inclusion and exclusion criteria can be enrolled and randomized.

#### Assessments during the treatment phase

During neoadjuvant treatment, patient evaluation for toxicity and adverse events is carried out on day 1 of each cycle and includes a physical examination, weight, vital signs, and hematologic and clinical chemistry laboratory tests. Patients receiving radiochemotherapy (Arm B) are assessed on a biweekly schedule with additional assessments on day 1, 15 and 29. Clinical restaging of the disease is performed preoperatively by esophagogastroduodenoscopy with endoscopic ultrasound and computed tomography of thorax, abdomen and pelvis. After completion of surgery, tumours are evaluated for histopathological response [16] and patients are monitored for perioperative morbidity and mortality as defined by adverse events occurring within 30 days after surgery. Before resuming treatment with adjuvant therapy and at day 1 of each cycle afterwards, patients are evaluated clinically including an electrocardiogram. After completing adjuvant therapy, a CT of thorax, abdomen and pelvis is performed only if any signs of tumour residue or relapse are suspected.

#### Assessments during follow-up

Clinical follow up visits will be performed every 3 months for up to 3 years according to the most recent German S3-guideline for the treatment of gastroesophageal cancer [4]. Afterwards, follow up visits will be performed every 6 months for up to 5 years, until death or end of follow-up. Assessment for relapsed disease includes a physical examination including body weight, vital signs and ECOG performance status as well as laboratory testing and computed tomography of chest, abdomen and pelvis. Toxicity and adverse events are recorded according to CTCAE version 4.03 until 90 days after the last study treatment and patients are assessed for quality of life during each visit. After first progression/relapse, no further staging is performed, and only survival status will be documented until end of follow-up.

### Statistical analysis

For the chemotherapy group a 3-year PFS of 40% is assumed [5]. An increase in the radiochemotherapy group to 55%, which translates to a hazard ratio of 0.65, is considered clinically relevant and achievable. The sample size is planned for an accrual period of 36 months and a maximum follow-up time of 60 months (follow-up from 24 to 60 months). Assuming exponentially distributed event times, a sample size of 306 patients (153 per group) will be necessary to detect a difference in event time distributions between both treatment groups under the given assumptions with a probability (power) of 80% on a two-sided level of significance of 5%. As about 10% of the patients are assumed to be lost to follow-up, the total number of patients included in the trial will be increased to 340 patients (170 per group). Patients dropping out will not be replaced.

Progression-free survival will be compared between both treatment groups with a log-rank test stratified by primary tumour site using a two-sided level of significance of 5%. Patients without an observed event of interest will be treated as censored observations. The primary analysis will be performed following the intention-to-treat (ITT) principle. Kaplan-Meier curves will be shown to illustrate survival functions for both treatment groups. Estimates for median survival and 1-, 3-, and 5-year survival with 95% confidence intervals (CIs) will be presented for both treatment groups. The hazard ratio with 95% CI will be estimated using a Cox regression model under the proportional hazards (PH) assumption with treatment group and tumour site included as fixed effects.

Secondary endpoints will be analysed in an explorative manner. R0 resection rate will be compared between both groups using chi-squared tests stratified for tumour site. For continuous outcomes (e.g. QoL scores) a linear regression model including treatment group and tumour site as fixed effects will be fit to the data. Analysis of overall survival will be performed as described for PFS.

Toxicities will be evaluated in the respective safety population. Absolute and relative frequencies of adverse events will be reported for each treatment group and for relevant subgroups as different tumour sites and institutions. Confidence intervals for probabilities of adverse events will be estimated. Group comparison will be conducted using Fisher’s exact test.

No interim analyses are planned. Efficacy parameters will be analysed at the end of the study unless the DSMB requests an unplanned interim analysis for safety reasons.

## Discussion

Incorporation of neoadjuvant treatment strategies in the management of locally advanced gastroesophageal adenocarcinoma has substantially improved clinical outcomes for this patient cohort [5–7]. One of the main advantages of neoadjuvant treatment is to improve the prognosis by reducing the size of the primary lesion with an increase in R0 resection rate, to reduce the number of infiltrated lymph nodes and to destroy microscopic tumour residuals [17]. Neoadjuvant treatment is indicated in patients with clinically staged T3 or resectable T4 carcinomas and those with suspicion of lymph node infiltration [4].

Despite these obvious successes, the overall prognosis remains unsatisfactory with 5-year overall survival rates still less than 50%. Intensive work has been done throughout the world to increase cure rates in patients with locally advanced GEJ cancers utilising chemotherapy and radiotherapy in the neoadjuvant and adjuvant treatment.

The currently available evidence has proven inconclusive to favour radiochemotherapy over chemotherapy alone in the neoadjuvant setting [13–15]. Published data on radiochemotherapy see the main advantage of radiochemotherapy in the higher local efficacy with improved R0 resection rates and high pathological complete response rates. However, it seems that the improvement of R0 rates and downstaging of disease following radiochemotherapy does not result in corresponding gains in long-term survival. The cause for this discrepancy is unclear but might be related to the lower control of distant relapse due to insufficient chemotherapy dosages. PFS and OS rates rather than local control rates therefore appear to be a more adequate surrogate for patient survival outcomes, particularly when comparing treatments. It is widely believed that patients with a high risk for distant failure gain benefit from induction therapy. In the context of locally advanced squamous cell carcinoma of the head and neck (LA-SCCHN), the addition of induction chemotherapy before concurrent chemoradiation resulted in a significant decrease in the distant metastasis rate (relative risk 0.58; *p* = 0.006) and higher complete response rates (relative increase 1.64; *p* = 0.010). However, this came at the cost of a greater level of therapy-related toxicities and did not translate into a significant survival benefit, as demonstrated by a large meta-analysis encompassing five randomized controlled trials with a total of 922 patients [18]. In colon cancer, the benefits of neoadjuvant induction chemotherapy are also being explored and recent data from the large randomized controlled FOxTROT trial (*n* = 1052) demonstrated a 50% reduction in incomplete resection rates and a borderline significant improvement of the 2-year-failure rate (HR = 0.77, *p* = 0.11) for those patients receiving neoadjuvant induction therapy before undergoing surgical resection [19]. Therefore, the RACE trial explores 2 cycles of induction therapy before oxaliplatin-based radiochemotherapy. Of note, cumulative oxaliplatin- and 5-Fluorouracil dosages will be roughly the same in the treatment arms.

Small prospective and retrospective trials [20] are subject to bias, particularly given that they often involve heterogeneous chemotherapy regimens which may no longer be the standard of care. The emergence of taxane therapy in FLOT-type regimens [7] has the potential to supersede older MAGIC-type chemotherapy, but its efficacy compared to chemoradiotherapy is unclear. Furthermore, the lack of adequately powered randomised trials has meant that a lack of clarity on the best choice of neoadjuvant treatment for esophageal cancer remains. Current treatment recommendations from major international societies including the European Society of Medical Oncology (ESMO) [21], National Comprehensive Cancer Network (NCCN) [22], and British Society for Gastroenterology (BSG) [23] are unanimous in recommending multimodal treatment but are not prescriptive for the regimen to be given. With the exception of very early stage tumours, all recommend either perioperative chemotherapy (CT) or neoadjuvant chemoradiotherapy (CRT). This has resulted in variable practice and treatment regimens [21–23]. Ongoing randomised studies such as the Australian TOPGEAR (which has reported interim feasibility but not survival data), German ESOPEC or UK-based Neo-AEGIS trials aim to provide better evidence to support one modality over the other; formal reporting of these trials is awaited [24–26].

The RACE trial sets out to overcome the limitations previous trials faced focusing on a clearly defined entity, i.e. adenocarcinoma of gastroesophageal junction, using the modern chemotherapeutic regimen FLOT as perioperative treatment and as induction therapy before radiochemotherapy within an adequate sample size to derive meaningful conclusions and ultimately guide clinical treatment strategies.

## Conclusion

The RACE trial is a multicentre prospective randomized controlled trial investigating the role of preoperative radiochemotherapy added to adequately dosed perioperative FLOT regimen compared to perioperative FLOT chemotherapy alone in patients with locally advanced adenocarcinoma of the gastroesophageal junction undergoing adequate oncological surgery. We hypothesize that addition of neoadjuvant radiochemotherapy to perioperative chemotherapy compared to perioperative chemotherapy alone will result in an increased progression-free survival.

## Data Availability

This article has used no dataset. Therefore, no additional data files are given.

## Abbreviations

AE: Adverse event
AIO: Arbeitsgemeinschaft Internistische Onkologie
AJCC: American Joint Committee on Cancer
AMG: Arzneimittelgesetz
ANC: Absolute neutrophil count
aPTT: Activated partial thromboplastin time
ARO: Arbeitsgemeinschaft Radiologische Onkologie
BSG: British Society for Gastroenterology
CF: Cisplatin / 5-fluorouracil
CR: Complete remission
CT: Computed tomography
CTCAE: Common terminology criteria for adverse events
CRT: Chemoradiotherapy
DSMB: Data safety monitoring board
ECF: Epirubicin / cisplatin / 5-fluorouracil
ECG: Electrocardiography
ECOG: Eastern cooperative oncology group
ECX: Epirubicin / cisplatin / Xeloda (capecitabine)
ESMO: European Society of Medical Oncology
FLOT: 5-fluororuacil / leucovorin / oxaliplatin
FLOT: 5-fluororuacil / leucovorin / oxaliplatin / docetaxel
5-FU: 5-fluorouracil
GCP: Good clinical practice
GEJ: Gastroesophageal junction
HR: Hazards ratio
INR: International normalized radio
ITT: Intention-to-treat
LA-SCCHN: Locally advanced squamous cell carcinoma of the head and neck
NCCN: National Comprehensive Cancer Network
QoL: Quality of life
ULN: Upper limit of normal
